# PAIRing Up Cargo Proteins

**DOI:** 10.1371/journal.pbio.1001334

**Published:** 2012-05-22

**Authors:** Charles Q. Choi

**Affiliations:** Freelance Science Writer, New York, New York, United States of America

**Figure pbio-1001334-g001:**
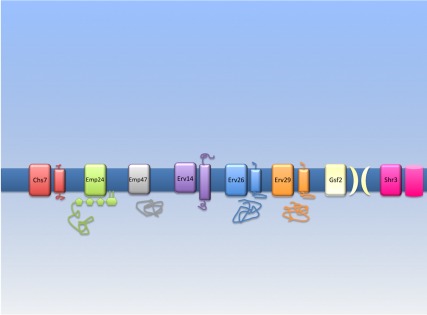
Towards a cellular “Traffickome.” Systematic analysis of cargo receptors (CRs) demonstrates the rules governing specificity versus promiscuity in ER exit.

[Fig pbio-1001334-g001]All newly made proteins destined to be secreted from our cells or to function at the cell surface—and many of those that function in membranes within the cell—start their lives in a convoluted heap of membranes within cells called the endoplasmic reticulum (ER). From there, they begin a journey that will take them through the stacked membrane compartments of the Golgi apparatus to their eventual destinations, transported from one compartment to another in small membrane vesicles. Proteins known as cargo receptors often perform the key function of selecting and tethering cargo proteins within the transport vesicles, but only a few such receptors have been identified to date, and which cargo proteins they pair with was mostly unknown. Now, Maya Schuldiner and her colleagues have devised a large-scale approach to discover these pairings between cargo proteins and cargo receptors, laying the groundwork to uncover the mechanisms that underlie this indispensable machinery.

The scientists call their approach “pairing analysis of cargo receptors”, or PAIRS. They start with yeast strains that have mutations in candidate cargo receptors—proteins that previous studies found were involved in traffic between the ER and the Golgi apparatus. A robotic system then systematically crosses these mutant yeast strains with a collection of other strains, each of which contains a potential cargo protein tagged with green fluorescent protein (GFP). The researchers then use an automated high-throughput microscope to help see which of the resulting strains have retained green fluorescence in the ER due to the cargo protein accumulating in the absence of its functional receptor. This strategy can be used to uncover the receptor for a specific cargo of interest.

They also show that this same methodology can be used to identify new cargo proteins for a known cargo receptor.

Schuldiner and colleagues mutated nine putative cargo receptors and followed the fates of 157 cargo proteins with their PAIRS technique. Knocking out these receptors affected 31 cargo proteins, 27 of which had not previously been linked to a particular cargo receptor. The spectrum of cargo uncovered for the cargo receptors helped define what specific cargo each receptor recognized. For example, past research hinted that all cargo for the receptor Erv26 were mannosyltransferases that function in the Golgi apparatus, but PAIRS suggests that Erv26 is specific for a subset of this group because the transport of several types of mannosyltransferase out of the ER was not affected by knocking out Erv26.

Perhaps the most striking finding was that the cargo receptor Erv14 interacts with a large number of cargo proteins. PAIRS showed that Erv14 is required for the trafficking of about a third of all plasma membrane proteins tested, including proteins with a wide array of functions and structures (permeases, transporters, multidrug transporters, lipid flippases, eisosome components, and proteins linked to cell polarity or cell wall regulation).

The numerous cargo proteins identified for Erv14 are all membrane-spanning proteins that reside late in the secretory pathway. Such proteins typically have longer transmembrane domains (TMDs) than other membrane-spanning proteins. When the researchers varied the length of the TMD of one of the cargoes bound by Erv14—Mid2—they found that the variants with the shortest TMDs had the most difficulty leaving the ER; in other words, Erv14 recognizes TMD length rather than sequence. Such a mechanism might permit a few receptors to transport a diverse range of cargo proteins without the need for multiple sequence-specific receptor motifs.

When Mid2 was fused to the tail of Sys1, a Golgi protein, it exited the ER rapidly, regardless of the length of its TMD, indicating that cargo proteins with a short TMD can be “rescued” by adding a different trafficking signal. Intriguingly, Mid2 variants with medium-length TMDs appeared to accumulate in the Golgi apparatus, hinting that TMD length might affect exit from the Golgi as well as the ER. This finding suggests the PAIRS approach might be used to shed light on the cargo sorting rules that apply to membrane protein traffic from the Golgi, as well as between other compartments.

Of the 157 cargo proteins the researchers analyzed, 126 were still able to exit the ER when any one of the nine candidate receptors was mutated, suggesting they may bind non-specifically to the proteins that coat the vesicles leaving the ER, or that they bind to cargo receptors that remain to be discovered. One of the next steps will be to uncover the many more cargo receptors that may exist in the more than 50 percent of yeast proteins that still have unknown function.

**Herzig Y, Sharpe HJ, Elbaz Y, Munro S, Schuldiner M (2012) A Systematic Approach to Pair Secretory Cargo Receptors with Their Cargo Suggests a Mechanism for Cargo Selection by Erv14. doi:10.1371/journal.pbio.1001329**

